# Future Teleworking Inclinations Post-COVID-19: Examining the Role of Teleworking Conditions and Perceived Productivity

**DOI:** 10.3389/fpsyg.2022.863197

**Published:** 2022-05-09

**Authors:** Clara Weber, Sarah E. Golding, Joanna Yarker, Rachel Lewis, Eleanor Ratcliffe, Fehmidah Munir, Theresa P. Wheele, Eunji Häne, Lukas Windlinger

**Affiliations:** ^1^Institute of Facility Management, Life Sciences and Facility Management, Zurich University of Applied Sciences, Zurich, Switzerland; ^2^Faculty of Health and Medical Sciences, School of Psychology, University of Surrey, Guildford, United Kingdom; ^3^Department of Organisational Psychology, Birkbeck University of London, London, United Kingdom; ^4^School of Sport, Exercise and Health Sciences, Loughborough University, Loughborough, United Kingdom

**Keywords:** COVID-19, remote working, home office, work privacy, productivity, teleworking

## Abstract

Organisations have implemented intensive home-based teleworking in response to global COVID-19 lockdowns and other pandemic-related restrictions. Financial pressures are driving organisations to continue intensive teleworking after the pandemic. Understanding employees’ teleworking inclinations post COVID-19, and how these inclinations are influenced by different factors, is important to ensure any future, more permanent changes to teleworking policies are sustainable for both employees and organisations. This study, therefore, investigated the relationships between the context of home-based teleworking during the pandemic (pandemic-teleworking conditions), productivity perceptions during home-based teleworking, and employees’ future teleworking inclinations (FTI) beyond the pandemic. Specifically, the study examined whether pandemic-teleworking conditions related to the job, and the physical and social environments at home, influenced employees’ FTI, and if perceptions of improved or reduced productivity mediated these relationships. Data were collected during April and May 2020 with a cross-sectional online survey of teleworkers (*n* = 184) in Germany, Switzerland, the United Kingdom, and other countries during the first COVID-19 lockdowns. Reported FTI were mixed. Most participants (61%) reported wanting to telework more post-pandemic compared to before the pandemic; however, 18% wanted to telework less. Hierarchical multiple regression analysis revealed that some teleworking conditions (job demands and work privacy fit) were positively associated with FTI. Other teleworking conditions (specifically, job change, job control, home office adequacy, and childcare) were not associated with FTI. Perceived changes in productivity mediated the relationship between work privacy fit and FTI. Findings highlight the role of work privacy fit and job demands in influencing pandemic productivity perceptions and teleworking inclinations post-pandemic. Results raise questions about the suitability and sustainability of home-based teleworking for all staff. As organisations plan to increase the proportion of teleworking post-pandemic, this study suggests there is a need to support employees who perceived their productivity to be poor while home-working during the pandemic.

## Introduction

Teleworking, while not a new phenomenon, has increased significantly during the COVID-19 pandemic (Milasi et al., 2021). Teleworking has multiple benefits for workers and organisations, e.g., greater work-life balance (Sullivan, 2012), increased flexibility and autonomy (Dambrin, 2004), and reduced overheads (Ferreira et al., 2021). Pre-pandemic, large-scale industry surveys (Global Workplace Analytics, 2015; Chartered Institute of Personnel Development [CIPD], 2020), and real estate research (Harris, 2016) indicated workers’ preference to telework more frequently. Research during the pandemic (Marzban et al., 2021; Naor et al., 2021; Tagliaro and Migliore, 2021) suggests that many would favour continued teleworking post-pandemic. However, this research predominantly focused on desires to change teleworking *frequency* rather than exploring underlying *motivations* for such desires. Further, how factors relating to the contextual conditions within which teleworking is undertaken (e.g., job design, social, and physical homework environment) might affect teleworking inclinations have been overlooked. A more nuanced approach to investigating teleworking inclinations could elicit greater insights into how best to implement and assist teleworking post-pandemic.

Research on this topic is conceptually and methodologically immature; however, some theoretical works from behavioural and management sciences offer approaches to studying teleworking inclinations. Whilst scholars do not put forward a distinct definition of teleworking inclinations (TIs) some (e.g., Baruch and Nicholson, 1997; Baruch and Yuen, 2000; Baruch, 2001) ground teleworking inclinations conceptually in the theory of reasoned actions (TRA, e.g., Fishbein and Ajzen, 1975) calling it “the Fishbein and Ajzen model for teleworking” (Baruch and Yuen, 2000, p. 525). This model states that values, norms, and behavioural beliefs precede attitudes; attitudes in turn “are antecedents to intentions or inclinations, which in turn, generate actions” (Baruch, 2001, p. 117). Unfortunately, teleworking inclination research has not yet tested the salience of the TRA in a teleworking context. Further, TI research appears underdeveloped. To date, the predominant focus lies on investigating predictors of teleworking attitudes (TAs); research on the attitude-inclination relationship or any other predictors of TI falls short. Baruch and Yuen (2000) identified predictors of TAs which include a combination of organisational/and personal values, norms, and behavioural beliefs related to productivity perceptions (“home work more effectively and efficiently”) and productivity-related issues arising from family commitments (“youngsters disturb my working process,” p. 532). Similarly, other researchers (Yap and Tng, 1990; Lim and Teo, 2000) identified TAs to be predicted by perceived advantages and disadvantages of teleworking, including productivity in/decreases but also context factors, such as home office setup/conduciveness, family commitments, and job design (increased autonomy). Lim and Teo (2000) concluded, “if individuals perceived an improved quality of work-life as a result of teleworking, they tend to have a more favourable attitude toward teleworking” (p. 577). This suggests that productivity increases and possible context factors facilitating productivity could be related to TAs, and as such, also to TIs.

However, TI research has further limitations: (1) it is inconsistent on whether predictors affect TIs or TAs and how TAs and TIs are differentiated, (2) it is inconsistent on how TAs and/or TIs relate to context factors, and (3) it is unclear how context factors, productivity perceptions, and TIs relate.

(1) For example, Lim and Teo (2000) included a TI item in their TA measure. Hence, identified predictors of TAs, such as productivity perceptions and context factors (home office setup, family commitments, and job design), could potentially predict TIs as well. Similarly, Baruch and Yuen (2000) positioned productivity perceptions as a predictor of TAs, according to their regression analysis. However, they also discussed that productivity perceptions (e.g., “work effective and efficient,” p. 533) can also “affect [participants’] decisions … to opt for teleworking” (p. 534), which they define as teleworking inclination.
(2) Other advantages and disadvantages that supposedly could affect the decision “to opt for teleworking” (TIs) include context factors, such as home office setup (e.g., “lack of space at home,” “equipment shortage,” “privacy”), work practices (“less supervision”) and family commitments (“more time with my family”; Baruch and Yuen, 2000). This suggests that these contextual factors impact TIs (and attitudes, see point 1). However, this relationship between contextual factors and TAs/TIs is inconsistent with Baruch and Nicholson’s overarching model of successful telework (“four factors of teleworking,” 1997, p. 27). The model categorises employees’ TAs and TIs as “the individual” dimension and positions it *alongside* three context factors “the home/work interface” (availability of physical facilities and child-care commitment), “the job” (job design aspects, such as control, task complexity, and technology requirements) and “the organisational culture.” As such, it is not yet clear if and *how* context factors and TIs interact; for example, if context factors interact hierarchically with TIs. We suggest that approaching this with a socio-ecological perspective (e.g., Sallis et al., 2015) could bring a number of benefits as this specifies multiple levels of influence on work behaviour in a hierarchically nested fashion (individual factors, social factors, built environment, and structural environment/job design/policy factors).
(3) Further, TA/TI research has associated perceived productivity and factors that facilitate productivity during telework with TAs/TIs. However, it is unclear on how context factors, perceived productivity and TAs/TIs actually relate. Other teleworking research, not focused on TA/TI, shows that productivity perceptions can be influenced by contextual factors (Gajendran and Harrison, 2007). And although Lim and Teo (2000) conclusion suggests a link between the improved quality of work-life as a result of teleworking and TAs, it remains unclear what the context factor-productivity perception-inclinations relationship is. Considering the prominence of productivity perceptions in prior TAs/TIs research and the mainstream suggestions (e.g., Deloitte, 2020; Gensler, 2020; Barrero et al., 2021) that teleworkers who experienced productive telework during the pandemic will likely remain in the home office post-pandemic, merits further research.

Hence, it can be concluded that (1) TI research is inconsistently grounded, (2) very little is known about predictors of TI, (3) the role of productivity perceptions and context factors are unclear and (4) it is unclear *how* these factors inter-relate.

Addressing these limitations and the empirical scarcity on the topic, this study (1) applies an established theoretical framework (socio-ecological framework), (2) investigates predictors of TI, and (3) the role of context factors, and the role of (4) productivity perceptions in predicting future teleworking inclinations (FTI). Besides addressing the empirical scarcity on the topic, a nuanced understanding of how contextual teleworking conditions might influence TIs can also help identify those who may not benefit from teleworking during and beyond COVID-19 because of their working conditions. This is essential knowledge for organisations that might encourage increased rates of teleworking to reduce office space expenditure (CBRE Research, 2020).

Therefore, the purpose of this study is to investigate if pandemic teleworking conditions and perceived changes in productivity during the pandemic influence post-pandemic FTI.

### Theoretical Approach to Investigating Context Factors: Socio-Ecological Framework

**FIGURE 1:**
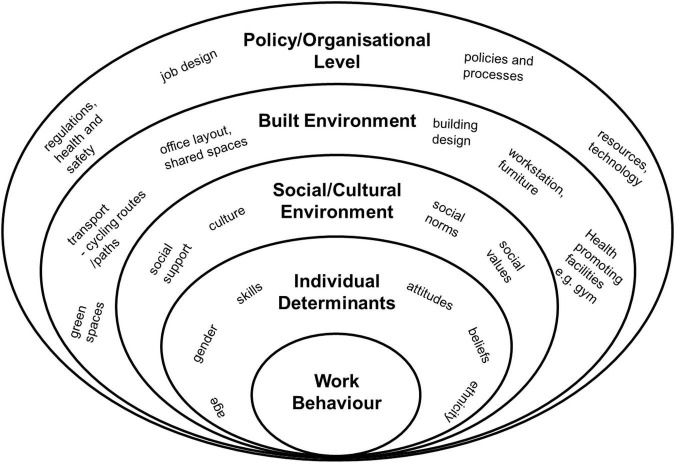
Ecological model adapted for teleworking (Sallis et al., 2015; Munir et al., 2021).

We examine pandemic teleworking conditions and their relationship to FTI through the theoretical lens of the socio-ecological framework on work behaviour (Sallis et al., 2015; Munir et al., 2021). As behavioural intentions are acknowledged to precede actual behaviour (e.g., TRA, Fishbein and Ajzen, 1975), we suggest this model could prove useful when investigating behavioural intentions, such as FTI. This theory-based framework suggests (health) behaviour at work is influenced by four nested hierarchical levels (see Figure 1): (1) individual determinants, (2) social environment, (3) built environment, and (4) the structural environment. At the first level, behaviour is influence by the individuals’ characteristics (e.g., gender, age, racial/ethnic identity, attitudes, and beliefs). At the second level, behaviour is influenced by social network and support systems that operates within that environment. We treat the second level as aspects of social family presence when teleworking, specifically family commitments. At the third level, behaviour is influenced by the built environment and its adequacy to meet the individuals’ work needs. We treat the third level as home office adequacy (including home office setup and privacy fit). At the fourth level, behaviour is influenced by structural factors, such as job design and teleworking policies. We treat the fourth level as job design during pandemic telework (job control, job demand, job change) in our study. Using the model in this context could complement the non-hierarchical model of “teleworking success” (also listing, “individual,” “job,” “home/work interface,” and “organisational culture”) by Baruch and Nicholson (1997) by nesting contextual factors (social, physical, and structural environment/job design) and relating them to behavioural intentions (oppose to teleworking success). Regarding the nesting-order of the levels, we approach the model from the outside-in starting with job pertinent factors. We start with the prerequisites of the job, specifically job design (structural environment), next we layer-in the physical environment, and finally we layer-in the social environment. Hence, this study assumes that pandemic teleworking conditions (social, physical, and structural environment/job design) have an impact on the wish to do more or less telework post-pandemic (FTI) in a hierarchical form. Furthermore, considering that prior teleworking attitude/inclination research hinted toward a relationship between context factors, productivity perceptions, and teleworking inclinations, the study will test the triangular relationship between these context factors/pandemic teleworking conditions (social, physical, and structural environment/job design), pandemic productive perceptions, and FTI.

### Pandemic Teleworking Conditions 1: Job Factors

Although teleworking inclination/teleworking attitude research positioned job factors, specifically job design aspects (such as control, task complexity, job demand, and technology requirements), as one of the four levels for successful telework (Baruch and Nicholson, 1997), little evidence is available on how job factors distinctly relate to TIs. Owing to empirical scarcity, we will build our argument on Lim and Teo (2000) conclusion that the overall quality of work-life experienced as a result of teleworking leads to favourable TAs. Hence, evidence from the early teleworking pandemic, which is not directly related to TIs but forms an overall pandemic teleworking experience, might also be of relevance and will be presented subsequently.

Findings from early teleworking research during the pandemic are equivocal on how job factors have impacted workers overall teleworking experience. Some teleworkers experienced reduced strain from job demands, better work outcomes (e.g., productivity), and more job resources (e.g., increased job control, Ipsen et al., 2020), while others experienced increased demands and reduced resources (e.g., decreased job control) in comparison to office working pre-pandemic (Chong et al., 2020). Hence, it is unclear if pandemic teleworking increases or decreases job demands and resources and how these are shaping the pandemic teleworking experience. Drawing from the fourth level of the socio-ecological framework (structural factors), this study focuses on job design, specifically job demands, job control, and job change.

*Job demands*, e.g., workload and responsibilities, are job conditions that require sustained cognitive and/or emotional effort, impede performance abilities (Bakker and Demerouti, 2007), and are associated with physiological and psychological costs (Bakker et al., 2014). Overall, job demands tend to be more important predictors than job resources (such as job control and job change) for shaping work experiences (Bakker et al., 2014). Pre-pandemic teleworking research reports that job demands can increase when colleagues are not immediately present to resolve work queries (Koroma et al., 2014). Similar notions, such as impeded information access or help from colleagues, have been only discussed (not tested) as a disadvantage of telework that could be related to TIs (reduced likelihood to opt for telework; Baruch and Yuen, 2000). Other than these reports, TA/TI research has not yet taken a position on whether job demands could be influential to these concepts. Pandemic research reports mixed results on whether job demands during teleworking are associated with positive or negative teleworking experiences. Some studies indicate that pandemic teleworking led to new pandemic-specific job demands (e.g., new teleworking-specific tasks or disruptive teleworking management tasks) associated with negative pandemic teleworking experiences (Chong et al., 2020). In contrast, other pandemic research suggests that teleworking during the pandemic is associated with increased efficiency due to a better work-environment fit in the home office (fewer distractions or interruptions), particularly for those who reported high job demand (Baert et al., 2020; Ipsen et al., 2020; YouGov, 2020). Considering that job demand is often the most important predictor for work experiences, and that little is known on if and how job demands and teleworking inclinations/FTI relate, we propose:

**H1.a**: Individuals reporting higher (vs. lower) job demand during the COVID-19 pandemic will have greater FTI.

*Job control* is defined as the perceived level of autonomy and influence workers have over when and how they work; examples of job control include autonomy in scheduling work, making decisions, and choosing working methods (e.g., Bakker and Demerouti, 2007). Pre-pandemic teleworking research indicated that teleworking is predominantly advantageous for job control, with teleworking enhancing perceived job control in terms of when and where work is done and how it fits around other aspects of life (e.g., Mann and Holdsworth, 2003; Dambrin, 2004; Gajendran and Harrison, 2007; Vander Elst et al., 2017). Although some pandemic studies echoed this notion and reported increased job control, which in turn was associated with positive teleworking experiences (Ipsen et al., 2021; Wang et al., 2021), others reported that job control has decreased during pandemic teleworking (Chong et al., 2020). Regardless of whether job control increased or decreased during pandemic teleworking, research suggests job control impacts the teleworking experience. In line with Lim and Teo (2000) suggestion that teleworking experience might inform TAs, and as such also TIs, an association between job control and TIs is likely. Further, prior TA/intention research identified increased job autonomy to be a teleworking advantage associated with positive TA (Yap and Tng, 1990); it is possibly also associated with positive TIs as of TA/intention item mixture (Lim and Teo, 2000). However, the relationship is still not distinctly studied. Therefore, we propose:

**H1.b**: Individuals reporting higher (vs. lower) levels of job control during the COVID-19 pandemic will have higher FTI.

*Job change* captures how well any organisational change is managed and communicated (Health Safety Executive [HSE], 2001). During the pandemic, organisational changes have been omnipresent (Amis and Janz, 2020). Sudden shifts toward primarily teleworking were largely unprepared for by organisations and employees (Bauer et al., 2020; Kaushik and Guleria, 2020), with over half of EU workers lacking any prior teleworking experience (Milasi et al., 2021). Well-managed job changes through high-quality supervision and managerial support (e.g., by providing ICT infrastructure or training; Milasi et al., 2021) represent a crucial job resource for teleworkers, likely to be critical for positive teleworking experiences during the pandemic. Teleworking attitude/intention research did not specify if or how job change and teleworking inclinations/FTI relate. Considering that job change appears important in informing the telework experience, particularly since the pandemic, alongside the empirical scarcity in TA/intentions research, we propose:

**H1.c**: Individuals perceiving job changes as more (vs. less) effectively managed by their employer during the COVID-19 pandemic will have greater FTI.

### Pandemic Teleworking Conditions 2: Environmental Factors

Baruch and Nicholson (1997) suggest that the home/work interface (including physical space and facilities) is one of four factors necessary for effective telework. Similarly, some teleworking inclination studies indicate that teleworking dis/advantages relating to home/work interface are associated with teleworking attitudes (Yap and Tng, 1990), and possibly teleworking inclinations (Lim and Teo, 2000). Since the home/work interface in the home office appears to have an important influence on pandemic teleworking experiences (Baert et al., 2020; Ipsen et al., 2020; YouGov, 2020), it may be associated with FTI. Specifically, this study explored two environmental factors: home office adequacy and work privacy fit.

*Home office adequacy* concerns the adequacy of furniture ergonomics (e.g., height-adjustable chair and desk), technology and workstation hardware (e.g., laptops, monitors, or telephones), and access to data and documents (cf. Hedge, 2016). These factors are resources that workers can draw on to complete their work. Theoretical and empirical TI research positions home office adequacy as a teleworking success factor (Baruch and Nicholson, 1997) and a significant predictor of TAs (“home environment that is not conducive for work”; “availability of a separate room for work,” Yap and Tng, 1990). Further, home office adequacy was discussed (not tested) to be potentially related to TIs (advantages “better work environment at home,” advantages “lack of space at home,” “equipment shortage”; Baruch and Yuen, 2000). Further, home office adequacy appears to affect teleworking experiences profoundly and, as such, could inform FTI. For example, adequacy has been found to be associated with job satisfaction and perceptions of productive work (e.g., Bosua et al., 2013). Home office inadequacy has been found to be associated with negative physical and psychological outcomes (including occupational stress, e.g., Sethi et al., 2011; techno-stress, e.g., Gaudioso et al., 2017; musculoskeletal health, e.g., Sauter et al., 1991; Derjani Bayeh and Smith, 1999; Sethi et al., 2011). Due to the unpreparedness for extreme teleworking during the pandemic (e.g., Milasi et al., 2021), home office inadequacy appears to be a common problem, evident in a reported lack of ergonomic furniture and adequate technology hardware, as well as inadequate access to documents and data (CBRE Research, 2020; Hofmann et al., 2020; Ipsen et al., 2020; Kunze et al., 2020). This may have hampered pandemic teleworking experiences (Pfnür et al., 2021) and as such, might impact FTI. Further, TA/TI research has indicated home office adequacy to be a potential predictor of TI. However, testing of this distinct relationship is still outstanding. Therefore, we propose:

**H1.d**: Individuals reporting greater (vs. lesser) adequacy of home office features (access to ergonomic furniture, technology hardware, and data/documents) will have greater FTI.

*Work privacy fit* addresses home office adequacy on a socio-spatial level, capturing the fulfilment of work privacy needs when considering distractions, interruptions, and task/conversation privacy (Weber et al., 2021). Privacy fit theory is rooted in P-E fit theory principles and as such suggests that work-related outcomes are maximised when environmental characteristics match individual needs (Weber et al., 2021). Empirical TA/TI research indicates that privacy elements are either predictive of TAs, possibly also TIs of methodological inattention (“youngsters disturb my working process”; Baruch and Yuen, 2000), or discussed to be potentially related to TIs (advantages “less distractions,” disadvantages “privacy”; Baruch and Yuen, 2000). Non-teleworking research indicates that work privacy fit impacts work experience (e.g., associated with various work- and health-related outcomes, satisfaction, work fatigue, self-rated productivity, cf. Weber et al., 2021; Weber and Gatersleben, 2021). Although privacy fit in teleworking contexts is under-explored, extant studies indicate that good privacy fit during teleworking is associated with a positive telework experience (e.g., silent home environments supporting concentrated work; Mirchandani, 1999; Montreuil and Lippel, 2003). In the context of COVID-19 teleworking, there is some evidence that teleworkers experience privacy-related advantages, such as better concentration, resulting in increased efficiency, greater productivity (Pfnür et al., 2021), and reduced occupational stress (e.g., Baert et al., 2020). Further, other research (Ipsen et al., 2020; Xiao et al., 2021; Wütschert et al., 2021) points to differences in work privacy fit at home across samples. For example, the item “getting distracted by people at home” was identified for some as an aspect that negatively affected telework during the pandemic, whereas for others the items entitled “getting time to focus on my work without interruptions by other people” and “don’t have anyone watch[ing] me” were positive aspects of telework in comparison to office work (Ipsen et al., 2020). This suggests that some pandemic teleworkers do not experience privacy fit during telework, which might have hampered their pandemic teleworking experience and, as such, might impact FTI. Further, TA/TI research has indicated privacy-related aspects to be potentially related to TIs. However, testing of this distinct relationship is still outstanding. Therefore, we propose that:

**H1.e**: Individuals reporting higher (vs. lower) levels of work privacy fit in their home workspace will have greater FTI.

### Pandemic Teleworking Conditions 3: Social Factors

*Childcare responsibility* has been positioned in theoretical and empirical TI research as teleworking success factors (home/work interface, Baruch and Nicholson, 1997; family commitments, Yap and Tng, 1990) and to be predictive of TAs, possibly also TIs as of methodological inattention (“youngsters disturb my working process”; Baruch and Yuen, 2000). In discussions, childcare responsibilities have been theoretically associated with TI (advantages “more time with my family”; Baruch and Yuen, 2000), however this was not tested yet. It is no surprise that in other non- TI teleworking research, family commitments have been identified as an important factor in seeking teleworking, offering flexibility over job and care responsibilities (Frone and Yardley, 1996). However, teleworking has also been acknowledged for its risk of blurring professional and domestic boundaries (Gajendran and Harrison, 2007), especially when care responsibilities must be accommodated and family intrusion frequently occurs during work time (Sullivan and Lewis, 2001). During nursery and school closures in the pandemic, many parents have combined work, childcare, and child education in the home, increasing burdens (e.g., Shockley et al., 2021). Hence, childcare is considered a social demand requiring additional management beyond job demands. Pandemic researchers point to potentially detrimental health impacts of pandemic telework amplified by multiplying non-work-related tasks/demands (e.g., childcare and home schooling), with negative effects on psychological wellbeing and work (Cox and Abrams, 2020; Shockley et al., 2021; Xiao et al., 2021). As such, the experience of teleworking during the pandemic is likely to be different for those with childcare responsibilities to those without; hence, childcare commitments are likely to influence teleworking experiences, and as such, potentially FTI. Further, considering indications of associations between family commitments and FTI, we propose:

**H1.f**: Individuals without (vs. with) childcare responsibilities will have greater FTI.

### Perceived Changes in Productivity

TAs/TI research is not clear on how context factors, productivity and TI relate and they did not investigate productivity as a distinct variable. However, productivity related predictors of teleworking attitudes were identified (‘home work more effectively and efficiently’, ‘youngsters disturb my working process’, Baruch and Yuen, 2000; ‘amount of work that can be done at home’, ‘home environment not conducive for work’, ‘distractions at home are hard to handle’ Yap and Tng, 1990). In discussions, productivity-related aspects have been theoretically associated with TIs (advantages “work effective and efficient [at home]”; Baruch and Yuen, 2000), but this has not been tested yet.

Pre-pandemic teleworking research echoes these advantages of teleworking, in that workers can experience improved productivity in the home office, attributed to fewer interruptions, longer working days, and work schedule flexibility (Gajendran and Harrison, 2007). There are mixed results regarding home working productivity during the pandemic, compared to working in the office (e.g., Marzban et al., 2021; Naor et al., 2021; Pfnür et al., 2021). However, most of these studies are descriptive, non-correlational studies, which fail to differentiate contextual factors associated with productivity gains or reductions. Contextual factors that include those related to the job, and physical and social environments, might explain these different teleworking experiences. For example, having job control and well-managed job changes can increase productivity (Bond and Bunce, 2003; Kerr et al., 2009). Environmental resources of home office adequacy (Pereira et al., 2019) and privacy fit appear associated with perceived productivity (Weber et al., 2021). Finally, associations between social demands (such as childcare) and reduced productivity during the pandemic have been reported (Shockley et al., 2021). Overall, studies that report work productivity gains during lockdowns, particularly those from real estate disciplines, are descriptive, non-correlational studies, which fail to differentiate workers with or without access to certain resources or the need to manage more demands (e.g., CBRE Research, 2020; Deloitte, 2020; YouGov, 2020). It can be assumed that having minimal social demands and access to sufficient job and environmental resources is associated with increased productivity. Based on prior teleworking and teleworking attitudes/inclination research, it firstly assumes that productivity perceptions are associated with FTI and secondly that productivity perceptions are associated with context factors. More specifically, it is assumed that individuals with specific pandemic teleworking conditions are likely to report greater FTI because they feel more productive in the home office. Hence, we propose:

**H2.a-e**: Changes in perceived productivity during pandemic teleworking (less/same/more productive than in the office) mediate relationships between predictors of FTI (job control, job change, home office adequacy, work privacy fit, and childcare) and FTI.

**FIGURE 2:**
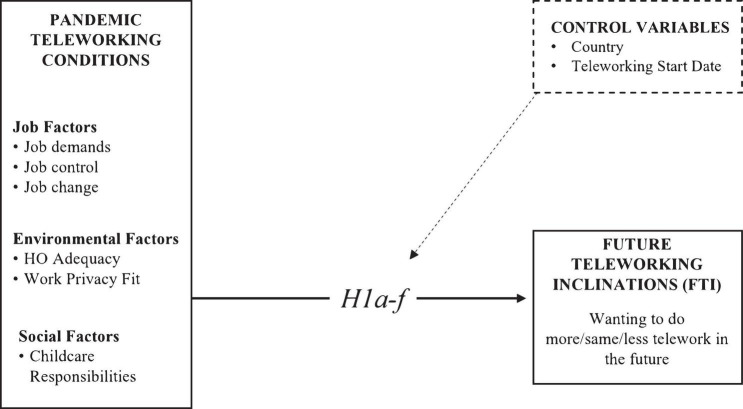
Hypothesised relationships H.1.

**FIGURE 3:**
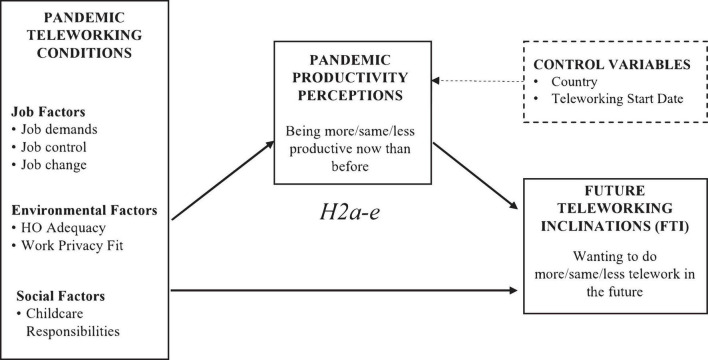
Hypothesised relationships H.2.

To summarise, the aim of this study was to investigate whether pandemic teleworking conditions (job, physical, and social environments) and productivity perceptions influence workers’ FTI beyond the COVID-19 pandemic. The hypothesised relationships are presented in Figures 2, 3.

## Materials and Methods

### Study Design and Procedure

An online, cross-sectional survey using the platform “Limesurvey” was conducted with an opportunistic sample of workers in Germany, Switzerland, the United Kingdom, and other countries (Australia, Austria, Belgium, Canada, Czech Republic, Denmark, Finland, France, Greece, India, Italy, Japan, Luxembourg, Netherlands, Portugal, Turkey, and Zimbabwe). The survey was administered in English to keep consistency across the countries. The survey was launched in mid/late March 2020, when strict social distancing measures had been in place from 18 to 26 days across the primary countries. Data used in this study were collected in a second wave of recruitment, 15 April–2 May 2020. Participants were recruited opportunistically *via* social media to recruit members of the public, and the researchers’ extended their own networks of colleagues, friends, and family *via* email. Inclusion criteria were that participants were employed, aged 18 years or older, and had primarily worked from home for at least 2 weeks prior to survey completion.

### Participants and Ethics

Participation was voluntary and participants provided informed consent. The survey was anonymous, and no identifying information was collected in accordance with regulations from Swiss federal law on human research. Data were treated confidentially, solely analysed for scientific purposes, and only shared with the research team. The data collection procedure and data use conformed with the Swiss Federal Data Protection Act, with all data stored on a secure university server. Participants were given a debrief page detailing links to healthcare providers and other sources of support relevant to COVID-19.

A total of 737 respondents participated, of which 258 were excluded due to illogical responses (illogical text in text fields) or extensive missing data (no responses apart from demographics). All cases with missing data were excluded,^1^ resulting in a sample of 479 respondents. As the item “perceived productivity” was added to the survey in the second of two recruitment waves (15 April), the final sample size of wave two, reported in this study, consists of 184 participants. Only the subsample of 184 participants was used in this study.

In this sample (*n* = 184), primary countries were almost equally represented (United Kingdom, 24.5%, Switzerland 26.6%, Germany, 36.4%); 12.5% of responses stemmed from “other countries.” The age distribution between participants was not equal, with 62.5% being female. Majority of participants (88%) fell in the age groups 21–30 (21.2%), 31–40 (46.7%), and 41–50 (20.1%). A third of the sample (33%) reported to have childcare responsibilities while pandemic teleworking; of those, most (97%) had one or two children. With regard to prior teleworking arrangements before the pandemic, 40.2% had teleworked from home before, on average 29.5% (*SD* = 23.52) per week. During the pandemic, participants worked on average 37.40 h (*SD* = 40.76) per week at home, which was for 45% about the same as before the pandemic (28.8% reported less than before; 26% reported more than before). Participant demographics are also provided in Table 1.

**TABLE 1:** Demographic details of the sample.

Characteristic		Count	Percentage (%)
**Country**			
	United Kingdom	45	24.5
	Switzerland	49	26.6
	Germany	67	36.4
	Other*	23	12.5
**Gender**			
	Female	115	62.5
	Male	69	37.5
**Age**			
	16–20 years	1	0.5
	21–30 years	39	21.2
	31–40 years	86	46.7
	41–50 years	37	20.1
	51–60 years	15	8.2
	61–70 years	6	3.3
**No. of children < 15 years**			
	0	126	68.5
	1	27	14.7
	2	29	15.8
	3	1	0.5
	4	1	0.5
**Childcare responsibilities**			
	Yes	62	33.7
	No	122	66.3
**Teleworked from home before pandemic**			
	Yes	74	40.2
	No	110	59.8

n = 184. *Other countries include: Australia, Austria, Belgium, Canada, Czech Republic, Denmark, Finland, France, Greece, India, Italy, Japan, Luxembourg, Netherlands, Portugal, Turkey, Zimbabwe.

An *a priori* power calculation with G*Power (to test H1 a.-f. including control variables), with a power (1-β) of 0.95, and α = 0.05. It was indicated that a sample of *n* = 86 would be required to detect large effects (*f*^2^ = 0.35), while a sample of *n* = 184 was necessary to detect moderate effects (*f*^2^ = 0.15); a sample of *n* = 1,304 would be required for the detection of small effects (*f*^2^ = 0.02). The target for recruitment was set at 300 (100 per primary country) to be able to control for potential confounding effects of the country and teleworking start date.

### Measures

Measures are described below. Descriptive statistics and correlations are provided in Table 2.

**TABLE 2:** Means, standard deviations, and correlations between study variables.

Variable		*M*	*SD*	1	2	3	4	5	6	7	8	9	10
1.	Telew. s.d. (Elapsed time)	30.5	9.5	–									
2.	Job demand	2.4	1.0	0.04	–								
3.	Job control	4.0	0.8	−0.02	−0.14	–							
4.	Job change	3.8	1.0	−0.03	−0.11	0.48**	–						
5.	HO adequacy—ergonomics	2.9	1.2	−0.08	−0.10	0.08	0.16*	–					
6.	HO adequacy—technology	3.7	1.0	−0.04	−0.16	0.19*	0.23**	0.48**	–				
7.	HO adequacy—D/D access	4.2	0.9	0.11	−0.10	0.02	0.20**	0.17*	0.45**	–			
8.	Work privacy fit	8.1	4.0	−0.04	−0.01	0.09	0.12	0.31**	0.22**	0.21**	–		
9.	Productivity	3.0	1.0	0.03	0.05	−0.10	−0.02	0.17*	0.08	0.14	0.35**	–	
10.	FTI	3.6	1.1	0.08	0.16*	−0.08	0.07	0.21**	0.14	0.17*	0.28**	0.37**	–

n = 184. As the item “perceived productivity” was added to the survey at a later stage, the sample for the correlation analysis was reduced. *p < 0.05, **p < 0.01 (2-tailed).

#### Demographics

Data were collected on age, gender, country of stay during the previous 2 weeks of lockdown, number of children <15 years,^2^ childcare responsibilities (caretaking and/or home schooling), teleworking start date, prior teleworking arrangements and the percentages, hours per week worked, if they had worked more or less since the pandemic.

#### Control Variables^3^: Country and Teleworking Start Date

The present study controlled for differences among countries as of variation in home office adequacy and teleworking preparedness (Milasi et al., 2021). It also controls for the date participants began working from home; a new variable “elapsed time” was created (teleworking start date subtracted by survey submission date).

#### Pandemic Teleworking Conditions 1: Job Demand, Job Control, and Job Change

Job demand, job control, and job change were assessed by the short version of the HSE indicator tool (Edwards and Webster, 2012). The dimensions of job demand (e.g., “I had unachievable deadlines”) and job control (e.g., “I had a say in my own work speed”) were each measured by four items. Job change was measured with three items (e.g., “Staff were always consulted about change at work”). Items were measured on a 5-point Likert scale, ranging from 1 (never) to 5 (always). Internal consistency for all three dimensions was acceptable (α_*jd*_ = 0.79; α_*jc*_ = 0.77; α_*jch*_ = 0.74). Mean composite scores were calculated. High scores reflect high levels of job demand, job control, and job change.

#### Pandemic Teleworking Conditions 2: Home Office Adequacy and Work Privacy Fit

Home office adequacy was assessed with three items (Ipsen et al., 2020): adequacy of (1) ergonomics, (2) technical equipment, and (3) access to data or documents. Participants were asked to rate whether the “home office was ergonomically [and] adequately set up,” “technical equipment in my home office was sufficient,” and “access to data or documents I had in my home office was sufficient.” Items were measured on a 5-point Likert scale, ranging from 1 (strongly disagree) to 5 (strongly agree). High scores reflect high home office adequacy.

Work privacy fit was measured using a simplified version of Weber (2019) Privacy at Work (PAW) inventory. Participants rated their satisfaction with the level of privacy they experience at work based on the importance of four separate dimensions of privacy assessment: (1) working without being overheard, (2) working without being overseen (being watched over by others), (3) working without being interrupted, and (4) working without distractions. Items were measured on a 5-point Likert scale, ranging from 1 (strongly disagree) to 5 (strongly agree). Internal consistency for privacy satisfaction and privacy importance was adequate (α_*ps*_ = 0.82; α_*pi*_ = 0.73). A composite score to reflect relative privacy fit was created by weighting privacy satisfaction ratings with privacy importance ratings using multiplication (cf. Lindner et al., 2016). High scores reflect high levels of privacy fit.

#### Perceived Changes in Productivity as of Pandemic Telework

Perceived changes in productivity were measured by one item developed for this study. Participants were asked “Overall, do you think you got more or less work done at home than if you had been working in the office?” The item was measured on a 5-point Likert scale, ranging from 1 (significantly less) to 5 (significantly more). High scores reflect perceptions of being more productive teleworking at home during lockdown than when working in the office before the lockdown (low scores, less productive at home).

#### Future Teleworking Inclinations

Future teleworking inclinations were measured by one item developed for this study. Participants were asked whether they would “consider doing more or less home office [work] than before when things return to ‘normal’.” This item was measured on a 5-point Likert scale, ranging from 1 (significantly less) to 5 (significantly more). The response option “not applicable” (NA) was included for workers not able to work from home due to company regulations/job specifications. NA responses were discounted from subsequent analysis. High scores reflect high inclinations to work more from home in the future than before the lockdown.

### Data Analysis

Data were analysed using IBM SPSS Statistics version 26.0 (IBM Corp., Armonk, NY, United States). Hypotheses 1.a-f were tested using a bootstrapped (10,000) hierarchical multiple regression model using the method of successive steps to separate the effects of control variables (country and elapsed time) and each context factor level (job, environmental, and social factors) on FTI. In the first step (1) country and elapsed time were introduced as control variables. The country variables data were dummy coded with the United Kingdom acting as the reference baseline. The remaining steps were: step 2, job factors (demand, control, and change); step 3 environmental factor 1 (home office adequacy); step 4, environmental factor 1 (work privacy fit); step 5, social factor childcare. Although regression analyses conducted without bootstrapping yielded the same effects, reporting bootstrapped results was preferred to increase the robustness of estimates of standard errors and to generate confidence intervals for regression coefficients (e.g., Fox, 2016). Hypotheses 2.a-e were tested using bootstrapped (10,000) mediation modelling using Hayes’s PROCESS tool (cf. Hayes, 2018). Testing H1 and H2 in separate models as opposed to one model was deemed appropriate because the analyses used observed rather than latent variables (cf. Hayes, 2018). Further, it should be pointed out that mediation analyses have been conducted, although correlational results (see Tables 2, 3) do not suggest direct total effects of some context variables on FTI. This aligns with the latest methodological understanding of mediation analysis, which positions a non-present direct total effect of X on Y that is not required for the detection of indirect effects; “the size of the total effect does not constrain or determine the size of the indirect effect” (cf. Hayes, 2018, p. 117). Taking a conservative approach, all variables, including control variables, were retained in the mediation models as covariates.

**TABLE 3:** Hierarchical regression model of job-related factors, environmental factors, social factors, and perceived productivity on FTI.

Variable	B [BCa] [BCa 95% CI]	SE B [BCa]	β	*t*	P [BCa]	*R* ^2^	*ΔR* ^2^
**Step1**					0.845	0.008	0.008
Teleworking start date (Elapsed time)	0.01 [−0.01 –0.03]	0.01	0.07	0.88	0.35		
Country_CH	−0.04 [−0.44 –0.34]	0.22	−0.03	−0.17	0.86		
Country_G	−0.11 [−0.59 –0.32]	0.24	−0.05	−0.50	0.66		
Country_Other	−0.10 [−0.65 –0.41]	0.30	−0.03	−0.33	0.74		
**Step2**					0.047	0.05	0.04
Teleworking start date (Elapsed time)	0.01 [−0.01 –0.02]	0.01	0.08	0.97	0.35		
Country_CH	0.09 [−0.30 –0.45]	0.22	0.04	0.37	0.70		
Country_G	0.04 [−0.40 –0.45]	0.24	0.02	0.17	0.87		
Country_Other	−0.05 [−.57 –0.44]	0.27	−0.02	−0.18	0.84		
Job demand	0.20* [0.02 –0.40]	0.08	0.17	2.15	0.01		
Job control	−0.18 [−0.41 –0.10]	0.13	−0.12	−1.42	0.16		
Job change	0.15 [−0.02 –0.33]	0.10	0.14	1.62	0.10		
**Step3**					0.006	0.12	0.07
Teleworking start date (Elapsed time)	0.01 [−0.01 –0.02]	0.01	0.07	0.96	0.37		
Country_CH	−0.17 [−0.60 –0.20]	0.22	−0.07	−0.67	0.48		
Country_G	−0.07 [−0.42 –0.26]	0.22	−0.03	−0.29	0.75		
Country_Other	−0.01 [−0.47 –0.47]	0.28	0.00	−0.02	0.10		
Job demand	0.21* [0.02 –0.42]	0.09	0.17	2.26	0.02		
Job control	−0.16 [−0.40 –0.13]	0.13	−0.11	−1.31	0.19		
Job change	0.10 [−0.11 –0.26]	0.09	0.09	1.03	0.28		
HO adequacy-Ergonomics	0.19* [0.06 –0.34]	0.07	0.20	2.41	0.01		
HO adequacy-Technology	0.05 [−0.17 –0.27]	0.10	0.04	0.47	0.61		
HO adequacy-D/D access	0.16 [−0.08 –0.40]	0.11	0.12	1.46	0.15		
**Step4**					0.003	0.16	0.05
Teleworking start date (Elapsed time)	0.01 [−0.01 –0.02]	0.01	0.08	1.02	0.30		
Country_CH	−0.17 [−0.57 –0.19]	0.22	−0.07	−0.67	0.49		
Country_G	−0.10 [−0.42 –0.20]	0.21	−0.04	−0.43	0.65		
Country_Other	0.01 [−0.52 –0.53]	0.30	0.00	0.04	0.97		
Job demand	0.19* [0.02 –0.38]	0.08	0.16	2.16	0.02		
Job control	−0.18 [−0.42 –0.11]	0.12	−0.12	−1.51	0.13		
Job change	0.09 [−0.09 –0.25]	0.09	0.08	0.99	0.28		
HO adequacy-Ergonomics	0.13 [−0.01 –0.28]	0.07	0.14	1.64	0.07		
HO adequacy-Technology	0.05 [−0.16 –0.26]	0.10	0.05	0.50	0.57		
HO adequacy-D/D access	0.11 [−0.11 –0.36]	0.11	0.08	1.00	0.33		
Work privacy fit	0.07** [0.03 –0.11]	0.02	0.23	3.05	0.002		
**Step5**					0.20	0.17	0.008
Teleworking start date (Elapsed time)	0.01 [−0.01 –0.02]	0.01	0.09	1.18	0.23		
Country_CH	−0.16 [−0.52 –0.15]	0.22	−0.06	−0.65	0.48		
Country_G	−0.13 [−0.45 –0.18]	0.21	−0.06	−0.59	0.53		
Country_Other	−0.03 [−0.57 –0.51]	0.30	−0.01	−0.11	0.89		
Job demand	0.19* [0.01 –0.37]	0.08	0.16	2.10	0.03		
Job control	−0.19 [−0.45 –0.11]	0.13	−0.13	−1.56	0.12		
Job change	0.09 [−0.10 –0.26]	0.09	0.08	1.01	0.30		
HO adequacy-Ergonomics	0.11 [−0.02 –0.28]	0.07	0.12	1.42	0.14		
HO adequacy-Technology	0.06 [−0.17 –0.27]	0.10	0.05	0.59	0.52		
HO adequacy-D/D access	0.11 [−0.10 –0.36]	0.11	0.08	1.01	0.33		
Work privacy fit	0.08** [0.03 –0.12]	0.02	0.27	3.32	0.002		
Childcare	0.26 [−0.09 –0.55]	0.20	0.10	1.29	0.19		

n = 184. *p < 0.05, ** p < 0.01. Bootstrap results are based on 1,000 bootstrap samples. BCa 95% CI = 95% bias-corrected and accelerated bootstrap confidence intervals. The dummy variable Country_UK was specified as reference category. The dummy variable No_Childcare was specified as reference category.

## Results

### Future Teleworking Inclinations

Most participants indicated wanting to telework more or significantly more post-pandemic than they did before the pandemic (*M* = 3.6, *SD* = 1.2, 61%, *n* = 113). However, 21% (*n* = 38) wanted to do the same amount of telework as they did before COVID-19, and 18% (*n* = 33) wanted to do less telework post-pandemic.

### Assessing Assumptions

The following assumptions for multiple regression were met (cf. Siegel, 2012): no outliers (Std. Residual Min = −2.70, Max = 2.20); no multicollinearity (max. correlation coefficients = 0.48; VIF Min = 1.1, Max = 1.9); independent errors (Durbin-Watson value = 2.02); approximately normally distributed errors (standardised residuals histogram and P-P plot); homogeneity of variance and linearity (standardised residuals scatterplot); non-zero variances (variance Min = 0.27, Max = 16.16); no biasing cases (Cook’s Distance values < 1).

### Hypotheses 1.a-f: Demands and Resources as Predictors of Future Teleworking Inclinations

**FIGURE 4:**
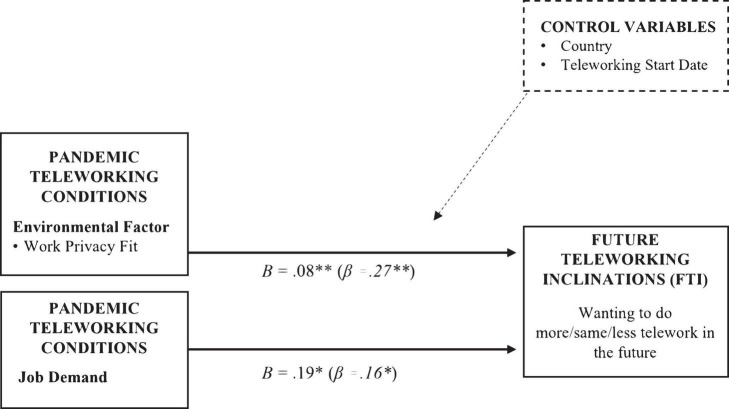
Supported hypothesised relationships. *p < 0.05; ^**^p < 0.01; *n* = 184. Non-significant predictors of FTI were job control (*p* =0 12), job change (*p* = 0.30), home office adequacy (ergonomic adequacy, *p* = 0.14; technical equipment, *p* = 0.52; data/document access, *p* = 0.033) and childcare responsibilities (*p* = 0.19).

A bootstrapped five-step hierarchical regression analysis was performed to explore associations between FTI and the predictor variables: job-related factors (H 1.a-c), home office adequacy (H 1.d), work privacy fit (H 1.e), and childcare (H 1.f). See Table 3 for results and Figure 4 for statistically significant relationships (*p* < 0.05).

Control variables (country and teleworking start date/elapsed time) were entered in block one. Model one was non-significant (*p* = 0.845) as were individual regression coefficients (range β = −0.03 –0.07; range *p* = 0.35 -0.86). Control variables account for only 0.8% of variance in FTI (*R*^2^ = 0.008); they remained non-significant predictors of FTI in all subsequent blocks.

To test H1.a-c, job-related variables were entered in block two. Model two led to significant improvements (*p* = 0.047), explaining an additional 4.4% of variance. Job demand (β = 0.17, *p* = 0.01) was significantly associated with FTI, whereas job change (β = 0.14, *p* = 0.10) and job control were not (β = −0.12, *p* = 0.16).

To test H1.d, home office adequacy variables were entered in block three. Model three led to a significant improvement (*p* = 0.006), explaining an additional 7% of variance. Ergonomic adequacy was significantly associated with FTI (β = 0.20, *p* = 0.01), whereas technical equipment (β = 0.04, *p* = 0.61) and data/document access (β = 0.12, *p* = 0.15) were not. Job demand remained a significant predictor in this model (β = 0.17, *p* = 0.02).

To test H1.e, work privacy fit was entered in block four. Model four led to a significant improvement (*p* = 0.003), explaining an additional 5% of variance. Work privacy fit was significantly associated with FTI (β = 0.23, *p* = 0.002). The effect of job demand remained (β = 0.16, *p* = 0.02), but the effect of ergonomic adequacy disappeared (β = 0.14, *p* = 0.07).

To test H1.f, childcare was entered in block five. Model improvement was non-significant (*p* = 0.20) as was the variable childcare (β = 0.10, *p* = 0.19); it accounted for 0.8% of variance. The significant effects of job demand (β = 0.16, *p* = 0.03) and work privacy fit (β = 0.27, *p* = 0.002) remained.

The final model (block five) explained 17% of the variance in FTI. As job demand and work privacy fit were significantly associated with FTI, Hypotheses 1.c and 1.e. are supported. As job control, job change, home office adequacy (ergonomic adequacy, technical equipment, and data/document access) and childcare either never had or lost their effect in later models, Hypotheses 1.a, 1.b, 1.d, and 1.f cannot be supported.

### Hypothesis 2.a-b: Productivity as Mediator of Job Resources-Future Teleworking Inclinations Relationship

**FIGURE 5:**
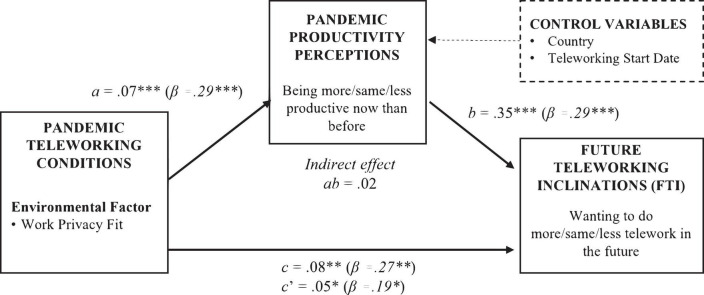
Supported hypothesised mediation. *p < 0.05; ^**^p < 0.01; ^***^p < 0.001; *n* = 184.

Two mediation analyses were performed to investigate H2.a-b that perceived productivity mediates the effect of job control and job change on FTI; see Figure 5 for significant relationships. None of the job-related variables were significantly related to perceived productivity (job control *a* = −0.17, *p* = 0.09; job change *a* = −0.01, *p* = 0.94). Perceived productivity predicted FTI while controlling for job control (*b* = 0.35, *p* < 0.001) and job change (*b* = 0.35, *p* < 0.001). The confidence interval for the indirect effect crossed zero in both cases; hence, there was no evidence of indirect effects of the job-related variables on FTI *through* perceived productivity (job control: *ab* = −0.06, SE = 0.044, LLCI = −0.16, ULCI = 0.018; job change: *ab* = −0.002, SE = 0.030, LLCI = −0.060, ULCI = 0.059). Direct (*c’* path) effects of job control (*c’* = −0.13, *p* = 0.27) and job change (*c’* = 0.09, *p* = 0.29) were not significant. Overall, these results do not support H2.a-b.

### Hypothesis 2.c-d: Productivity as Mediator of Environmental Resources-Future Teleworking Inclinations Relationship

Mediation analyses were performed to investigate if perceived productivity mediated the effect of home office adequacy variables (2.c) and work privacy fit (2.d) on FTI.

None of the home office adequacy variables were significantly related to perceived productivity (ergonomics *a* = 0.09, *p* = 0.20; technology *a* = −0.06, *p* = 0.51; data/document access *a* = 0.08, *p* = 0.41). Perceived productivity predicted FTI while controlling for home adequacy variables (*b* = 0.35, *p* < 0.001). There was no evidence of an indirect effect of the home office adequacy variables on FTI *through* perceived productivity (ergonomics *ab* = 0.03, SE = 0.025, LLCI = −0.013, ULCI = 0.084; technology *ab* = −0.02, SE = 0.030, LLCI = −0.085, ULCI = 0.035; data/document access *ab* = 0.03, SE = 0.041, LLCI = −0.052, ULCI = 0.115). Direct (*c’* path) effects of home office adequacy variables on FTI were non-significant (ergonomics *c’* = 0.08, *p* = 0.28; technology *c’* = 0.08, *p* = 0.42; data/document access *c’* = 0.08, *p* = 0.43). Overall, these results do not support H2.c.

Work privacy fit was positively related to perceived productivity (*a* = 0.07, *p* < 0.001); i.e., the better participants rated their work privacy fit at home, the better they evaluated their productivity. Perceived productivity predicted FTI while controlling for work privacy fit (*b* = 0.35, *p* < 0.001); i.e., the better participants evaluated their productivity, the more likely they were to express FTI. There was evidence of an indirect effect of work privacy fit on FTI *through* perceived productivity (*ab* = 0.02, SE = 0.090, LLCI = 0.009, ULCI = 0.043); i.e., having good work privacy fit indirectly influenced FTI *through* its effect on experiencing greater perceived productivity in the home office. The direct effect of work privacy fit on FTI of *c’* = 0.05 was statistically significant (*p* = 0.02); i.e., having good work privacy fit influenced the inclination to increase teleworking in the future independent of perceived productivity effects. These results support H2.d.

### Hypothesis 2.e: Productivity as Mediator of Social Demands-Future Teleworking Inclinations Relationship

Mediation analysis was performed to investigate H2.e that perceived productivity mediates the effect of childcare responsibilities on FTI. Having childcare responsibilities was not significantly related to perceived productivity (*a* = −0.22, *p* = 0.20). Perceived productivity significantly predicted FTI while controlling for childcare (*b* = 0.35, *p* < 0.001). There was no evidence of an indirect effect of childcare on FTI *through* perceived productivity (*ab* = −0.08, SE = 0.065, LLCI = −0.219, ULCI = −0.040). The direct effect of childcare on FTI (*c’* = 0.34, *p* = 0.09) was statistically significant. Overall, this does not support H2.e.

## Discussion

This cross-sectional study examined why pandemic workers might wish to telework more or less post-pandemic (future teleworking inclinations; FTI). Specifically, it examined which contextual pandemic teleworking conditions influenced FTI, and whether this is due to differing perceptions of productivity at home vs. the office.

### Predictors of Future Teleworking Inclinations: Job Demand, Work Privacy, and Productivity Perceptions

Overall, most participants in this study reported wanting to telework more post-pandemic. This was especially true for workers that experienced higher levels of job demand and that had greater work privacy fit in their home office. As job demand was the strongest predictor of FTI, teleworking may afford greater resources to cope with job demand, for example, due to the time gained by not commuting and/or having fewer distractions at home (Baert et al., 2020; Naor et al., 2021). Work privacy fit also predicted FTI; a relationship that was mediated by productivity perceptions. This could suggest that participants who experienced greater work privacy at home were more likely to want to telework more post-pandemic (FTI) because it made them feel more productive. However, as of the cross-sectional design of the study, the direction of the meditation remains unclear (e.g., O’Laughlin et al., 2018). The partial mediating effect indicates that work privacy fit explains some variance in FTI independently of perceived productivity improvements. As such, work privacy fit may also be associated with other determinants of teleworking attitudes besides productivity, e.g., health-related aspects (e.g., Laurence et al., 2013; Weber et al., 2021), which in turn might influence the desire for more teleworking post-pandemic. However, as directional effects were not tested, this remains speculation.

### Unanticipated Findings: Context Factors Without Effects

Not all findings were as hypothesised. First, job control had no observed effect on FTI or perceived productivity. This is surprising as prior research indicates increased control during teleworking (e.g., Gajendran and Harrison, 2007) and productivity-control associations (Bond and Bunce, 2003). This lack of observed effect may be explained by control being more immanent to the job than to teleworking inclinations. Further, other moderating work-related factors beyond the scope of this study might also explain this effect, e.g., daily task setbacks related to pandemic management (see Chong et al., 2020). Second, job change was not observed to predict FTI, which is surprising as prior research indicates its association with teleworking experiences and productivity (Kerr et al., 2009). Rapid changes in both work and home environments due to the pandemic may have dampened the effect of job change. Third, the environmental resources of adequate ergonomics, technology, and document/data access (home office adequacy) had no observed effect despite their identified importance considering job satisfaction (Loghmani et al., 2013), productivity perceptions (Bosua et al., 2013; Pfnür et al., 2021), and health (Pereira et al., 2019). Ergonomics became a non-significant predictor after work privacy fit was added to the regression model. This could be explained by lack of power as the model grew, or by work privacy fit covering aspects of the home office setup. Finally, childcare responsibility had no observed effect on FTI, despite previous pandemic research indicating associations with poor work outcomes and mental health (e.g., Cox and Abrams, 2020; Shockley et al., 2021; Xiao et al., 2021). Non-significant associations could be explained by associations between childcare and work privacy fit, which both analyses (regression and mediation) controlled.

### Implications for Teleworking Research

This study makes several contributions to teleworking research. First, it adds to previous teleworking inclination research by taking a nuanced view on predictors of TI, specifically contextual factors. Secondly, to study the effect of contextual factors on FTI, it applies a hierarchically nested socio-ecological framework on work behaviour and relates these contextual factor levels to behavioural intentions. This framework complements Baruch and Nicholson (1997) model on teleworking success, which used similar context factor levels but did not specify *how* these levels relate to an overarching construct (teleworking success). Third, previous research was unclear about the role of productivity perceptions in informing TIs, which this study discretely investigated. Fourth, it investigates the triangular relationship between FTI, context factors, and productivity perceptions. Finally, it indicates that job demand and the physical environment/privacy fit effects account for FTI *via* an effect on perceived productivity increase/decrease. These contextual factors predicted perceived performance increases/decreases during pandemic teleworking, and this was related to FTI.

### Implications for Organisations

The study has practical implications as teleworking is being promoted as an option to reduce office space expenditure post-pandemic (CBRE Research, 2020). Understanding predictors of FTI and productivity can help organisations form teleworking strategies that better match workers’ different capabilities, rather than applying one policy to all staff. Considering that teleworking might not match all individuals’ capabilities and preferences, the universal application of teleworking is not advised, and some may benefit from only limited teleworking or require additional support. This notion is supported by pre-pandemic research (e.g., Baruch and Yuen, 2000) but also by emerging pandemic research pointing to productivity reductions for large amounts of workers (Pfnür et al., 2021). Given the role of perceived productivity in predicting FTI, and given the established effects of peer, managerial, and organisational support on perceived productivity during telework (Bentley et al., 2016), there is value in ensuring that workers are provided with job resources to increase actual and perceived productivity. Organisations could undertake profiling to understand who can telework effectively (considering home office conditions and task profile) and who needs additional support. This can help determine an optimal teleworker/office worker balance, facilitating workforce well-being and performance (Ipsen et al., 2021) and inform the development of longer-term teleworking policies that are optimal and sustainable for both employees and organisations during the remainder of, and beyond, the COVID-19 pandemic.

## Limitations

Possible limitations relating to the methodology should be acknowledged. First, convenience sampling inevitably risks representativeness; for example, 63% of the sample were female and only 11% were aged 51 or above. Second, it should be noted that participants outside the three primary countries were grouped together when analysing potential country differences. Although this group included participants from diverse countries, it was deemed appropriate to group them together for this analysis given that the focus of the study was on teleworking. Whilst acknowledging potential differences between countries grouped into “Other countries,” some similarities can still be assumed in relation to the experience of teleworking during the pandemic (such as the presence or absence of adequate space and equipment at home, childcare responsibilities, and privacy fit). Third, the investigation of productivity perceptions could have been further differentiated (e.g., work content execution or effectiveness) so that future research could explore various aspects of productivity in more detail. Relatedly, the one-item measures for “perceived changes in productivity” and “future teleworking inclinations” bare the risk of reduced reliability and validity (e.g., Gardner et al., 1998). However, no multi-item measure for FTI/TI is available to date, with prior studies having resorted to using single items (Baruch and Yuen, 2000). Furthermore, self-reported measures, particularly on productivity but also on equipment adequacy, are susceptible to response biases, such as social desirability (e.g., Paulhus, 1991; Kuncel and Tellegen, 2009). Although this is a persistent limitation in organisation research, manifold techniques are available to minimise or control this risk (e.g., Jordan and Troth, 2020). Finally, the cross-sectional design examined variables at one single moment in time and prevents causal inferences. Relatedly, cross-sectional mediation analyses carry the risk of misrepresentation of psychological processes and ambiguity of the direction of the effect; longitudinal mediation models may provide better representations of mediation processes (e.g., O’Laughlin et al., 2018), and future research could look to examine variables from this study longitudinally.

The socio-ecological framework could provide a useful lens through which to identify relevant individual and contextual factors and to investigate their relationship to overarching behavioural constructs. However, it is possible that specific individual factors, social factors, the built environment, and the structural environment/job design/policy factors were unduly represented in our study. This merits further research with a fuller reflection of all levels. For example, data were not collected about sectors, occupation, company size, self-employment, tasks or roles; across these factors, workers may differ in their experiences of teleworking, alongside their teleworking infrastructure pre (Milasi et al., 2021) and during the pandemic (Ipsen et al., 2020). Further, other than family commitments, this study did not account for wider aspects of the social environment, such as social support systems from co-workers, managers (e.g., Chong et al., 2020), and family. Finally, the study did not assess the participants’ prior teleworking conditions, attitudes, or inclinations, which might influence FTI. Therefore, conclusions cannot be drawn over whether factors such as job control, job demand, and privacy fit, changed compared to pre-pandemic experiences. However, a full investigation of the context levels was beyond the scope of this study. The study did, however, investigate potential response differences due to varying teleworking starts and prior teleworking arrangements^3^, which were non-significant.

## Conclusion

This study extends previous teleworking research in two ways: first, by exploring the impact of pandemic teleworking conditions on FTI and teleworking conditions-attitude-inclinations relationships and second, by adapting the socio-ecological framework for telework. It revealed that those with higher job demand and better work privacy are more likely to want increased levels of teleworking post-pandemic because they perceived increases in their productivity while pandemic-teleworking. Those without adequate work privacy did not want to increase teleworking post-pandemic because they perceived reductions in their productivity. These findings point to different capabilities for post-pandemic teleworking due to differing home office conditions. This study offers a nuanced approach to the investigation of teleworking inclinations and can inform strategies on how to best implement teleworking post-pandemic to ensure any future, more permanent changes to teleworking policies are optimal and sustainable for both employees and organisations.

## Data Availability Statement

The data for this study will not be made publicly available, as participants did not consent for this possibility at the time of recruitment. The raw data supporting the conclusions of this article can be made available by the authors upon request.

## Ethics Statement

Ethical review and approval was not required for the study on human participants in accordance with the local legislation and institutional requirements. The patients/participants provided their written informed consent to participate in this study.

## Author Contributions

CW, SG, and JY contributed to conceptualisation and writing – review and editing. EH and CW contributed to data curation. CW contributed to formal analysis, visualisation, and writing – original draft preparation, supervision, and project administration. All authors contributed to conceptualization, methodology, investigation, resources, and writing – review and editing.

## Conflict of Interest

The authors declare that the research was conducted in the absence of any commercial or financial relationships that could be construed as a potential conflict of interest.

## Publisher’s Note

All claims expressed in this article are solely those of the authors and do not necessarily represent those of their affiliated organizations, or those of the publisher, the editors and the reviewers. Any product that may be evaluated in this article, or claim that may be made by its manufacturer, is not guaranteed or endorsed by the publisher.

## Footnotes

1 No cases had missing values for the units of analysis for composite scores (total scores on each measure). A total of 273 (57.0%) cases had missing data at the level of individual items on various scales. Of all possible item values, 10.09% (1,209/11,975 items) were missing. Little’s Missing Completely at Random Test indicated that the data in question were missing completely at random and not systematically, χ^2^(369, *n* = 479) = 407.56, *p* = 0.08.

2 The age limit of 15 years was deemed appropriate as the age of legal responsibility and liability, as well as maturity concerning various aspects ranges between 14 and 16 years in the primary countries (10–16 years in Switzerland; 14 years in Germany; 10–16 across the United Kingdom; CRIN, 2020). Hence, it was assumed that children from the age of 15 years onward would require less supervision than younger children.

3 We controlled for further variables such as gender, age, or prior teleworking arrangements. Preliminary analyses indicated that there was no difference in FTI or productivity distribution by age groups [e.g., FTI *F*(5, 178) = 1.01, *p* = 0.57], gender [e.g., FTI *t*(182) = −0.12, *p* = 0.13] or prior teleworking arrangements [e.g., FTI *t*(180) = −0.41, *p* = 0.18]. Hence, this variable was not included for further analyses to ensure it does not unduly reduce power.
